# Thyroid hormones as biomarkers of lung cancer: a retrospective study

**DOI:** 10.1080/07853890.2023.2196088

**Published:** 2023-04-04

**Authors:** Zhenchao Ma, Pengtang Song, Dongxiang Ji, Mingjia Zheng, Guoqing Qiu, Zhicong Liu, Bin Wang

**Affiliations:** a Department of Radiation Oncology, Huzhou Central Hospital, Affiliated Central Hospital Huzhou University, Huzhou, People’s Republic of China; b Department of Pathology, Huzhou Central Hospital, Affiliated Central Hospital Huzhou University, Huzhou, People’s Republic of China; c Department of Respiratory Medicine, Huzhou Central Hospital, Affiliated Central Hospital Huzhou University, Huzhou, People’s Republic of China

**Keywords:** Lung cancer, thyroid hormones, diagnostic biomarker, free triiodothyronine

## Abstract

**Background:**

Thyroid hormones are key regulators of several physiological processes, including differentiation, embryonic development, proliferation, and metabolism. Several prospective studies have shown a relationship between hyperthyroidism and cancer incidence; however, since the association between thyroid hormone levels and lung cancer remains controversial, this study aimed to determine the correlation between the same.

**Methods:**

We retrospectively analyzed 289 patients, who were diagnosed with lung cancer at the Huzhou Central Hospital between January 2016 and January 2021, and 238 healthy subjects. The baseline clinical data of two groups were collected. The concentrations of thyroid hormones, tumor CEA, CYF, SCC, and NSE in both the lung cancer patient and healthy volunteer groups were analyzed. Student’s *t*-test or Mann–Whitney test was used to compare continuous variables. A chi-square test was adopted to estimate the relationship between serum thyroid hormones level and clinical characteristics of lung cancer cases. ROC curve analyses were used to determine the characteristics of thyroid hormones for recognizing lung cancer.

**Results:**

The results showed that serum thyroid stimulating hormone (TSH), total thyroxine, total triiodothyronine, and free triiodothyronine (FT3) levels were significantly decreased, while free thyroxine (FT4) levels were increased in patients with lung cancer. In addition, FT3 was identified as a potential diagnostic biomarker of stage I–IV lung cancer with the area under the curve values of 0.807. What’s more, FT3 and FT4 were used in combination with CEA and were identified as potential diagnostic biomarkers of stage 0 lung cancer (Tis) with the area under the curve values of 0.774.

**Conclusions:**

Our study highlights the possibility of using thyroid hormones as innovative diagnostic markers for lung cancer.

## KEY MESSAGES

1. Serum TSH, TT4, TT3, and FT3 levels were significantly decreased in patients with lung cancer.
2. low TT3 concentration was positively associated with age (*p* < 0.05), sex (*p* < 0.05), tumor size (*p* < 0.05) and lymph node metastasis (*p* < 0.05). Moreover, the concentration of FT3 was dependent on age (*p* < 0.05) and tumor size (*p* < 0.05). The serum FT4 concentration was discernible with obviously higher concentration in stage IV patients compared with stage I–III patients (*p* < 0.05).
3. When FT3 was used in combination with CEA and CYF, the sensitivity and specificity in the screening for stage I–IV lung cancer were markedly increased to 85.9% and 97.5%, respectively. When we included FT3, FT4, and CEA in the diagnosis, the AUC was 0.774. The sensitivity and specificity of screening for stage 0 lung cancer were increased to 70.2% and 75.2%, respectively.

## Introduction

Lung cancer remains the second most common cancer in the United States and continues to rise globally [1]. Despite improvements in drug therapy, including chemotherapy targeted therapy and immunotherapy, lung cancer is the leading cause of cancer-related death in men and women [2]; one of the reasons stated in the literature for high mortality is that a large proportion of patients are diagnosed at an advanced stage [3]. Based on the Tumor Node Metastasis (TNM) staging system, 5-year survival estimates in non-small cell lung cancer ranges from 73% in stage IA to 13% in stage IV disease [4]. Hence, the detection of lung cancer at an early stage is of great importance for increasing the disease-free survival (DFS) and overall survival (OS) of patients [5]. Although early screening of lung cancer by low-dose computed tomography resulted in a 20% mortality reduction, body fluid-based biomarker analysis is still more convenient [6]. Carcinoma embryonic antigen (CEA), CYFRA21-1 (CYF), squamous cell carcinoma (SCC) antigen, and neuron specific enolase (NSE) are extensively used tumor biomarkers of lung cancer and play an important role in surveillance during disease treatment. However, their sensitivity and specificity are unsatisfactory due to benign lung diseases, such as pulmonary fibrosis [7]. Thus, new noninvasive biomarkers for the detection of lung cancer are urgently required.

Thyroid hormones are iodinated endogenous compounds necessary for the regulation of multiple cellular activities, such as cell differentiation, growth, and metabolism [8]. Recent epidemiologic studies have shown a relationship between thyroid hormone concentrations and cancer risk [9]. These studies yielded conflicting results. Hyperthyroidism is associated with a higher risk of pancreatic cancer [10], lung cancer [11], breast cancer [12] and prostate cancer [13]. In contrast, Schmidinger M et al. found that progress was observed in renal cell carcinoma patients with hypothyroidism due to tyrosine kinase inhibitors [14]. Furthermore, data regarding abnormal thyroid function and cancer risk do not provide the diagnostic value of thyroid hormones in lung cancer. Hence, in the current study, we not only assessed the association between thyroid function and lung cancer, but also investigated the diagnostic value of thyroid hormones in lung cancer.

## Materials and methods

### Study population

We included 289 patients who were diagnosed with lung cancer at Huzhou Central Hospital, Affiliated Central Hospital Huzhou University, between January 2016 and January 2021 for the retrospective analysis. The patients had not received any local or systemic anticancer treatment before diagnosed. Of the lung cancer patients group A who were diagnosed with stage I–IV, 159 were male and 46 were female. TNM staging was unified based on the eighth edition of IASLC (the International Association for the Study of Lung Cancer system) for lung cancer. The median age of the subjects was 66 years (range: 30–91 years). While, in lung cancer patients group B, 41 males and 43 females (stage 0) were included in the study. Malignancy was confirmed independently based on pathologic specimens reviewed by two pathologists. Meanwhile, 238 health examinees in Huzhou Central Hospital, Affiliated Central Hospital Huzhou University during January 2018 and January 2021 were taken as the control group. Health examinees with lung disease, including pulmonary nodules or thyroid disease affecting thyroid hormone metabolism, were excluded. We also excluded patients with a history of cancer, except for lung cancer. These patients were age- and sex-matched to the control group. The information of the patients and healthy individuals is recorded and summarized in Table 1. The study protocol was approved by the Institutional Review Board of Huzhou Central Hospital, Affiliated Central Hospital Huzhou University, and all patients signed an informed consent form when they entered the hospital for examination.

**Table 1.** Clinicopathological data of all patients and healthy subjects included in the study.

Variables	Healthy subjects	Lung cancer patients A	Lung cancer patients B	
Athnicity	Asians	Asians	Asians	
Age				
Mean ± SD	65.09 ± 0.700	66.82 ± 0.655	65.77 ± 0.181	
Sex				
Female	55	46	43	
Male	183	159	41	
Tumor type				
SCLC		51	1	
NSCLC		154	83	
Tumor size (maximum diametercm)				
≤4 cm		93	84	
>4 cm		112	0	
Lymph node metastasis				
Positive		179	0	
Negative		26	84	
TMN stage				
0		0	84	
I/II/III		81	0	
IV		124	0	

### Laboratory measurements

Thyroid function markers, thyroid stimulating hormone (TSH), total thyroxine (TT4), total triiodothyronine (TT3), free thyroxine (FT4), and free triiodothyronine (FT3) were assessed in the leftover blood samples collected from the patients. The assessment of tumor CEA, CYF, SCC, and NSE were routine diagnoses in the patients. The thyroid function markers and tumor CEA, CYF, SCC, and NSE were routine physical examinations in health examinees. The normal range of markers is as follows: TSH, 0.35–4.94 µIU/mL; TT4, 3.37–11.72 µg/dL; TT3, 0.58–1.59 ng/mL; FT4, 0.70–1.48 ng/dL; FT3, 1.58–3.91 pg/mL; CEA, 0–5.00 ng/mL; CYF, <3.30 ng/mL; SCC, 0–1.50 ng/mL; NSE, <15.20 ng/mL. All laboratory measurements were performed once the patients were diagnosed with lung cancer and before any local or systemic anticancer treatment.

### Statistical analysis

Continuous data were summarized as mean ± SD. Statistical analyses were performed using SPSS (Statistical Package for the Social Sciences) software (version 19.0; IBM, Chicago, IL, USA), and a *p* value of 0.05 was considered statistically significant. The Kolmogorov-Smirnov test evaluated the distribution characteristics of continuous data. Student’s *t*-test or Mann–Whitney test was conducted to assess the differences between the control and lung cancer groups. Pearson’s *χ*^2^ associations between thyroid function markers and patient clinicopathological parameters were assessed.

Furthermore, to evaluate the diagnostic value of thyroid function markers, the receiver operating characteristic (ROC) curve was plotted using Medcalc 19.2 (MedCalc bvba, Ostend, Belgium), and relevant results were estimated using the area under the curve (AUC). The corresponding sensitivity and specificity were calculated at different markers cut-offs when the Youden Index is equal to 1.

## Results

### Baseline characteristics of healthy and lung cancer cohorts

**Figure 1.**
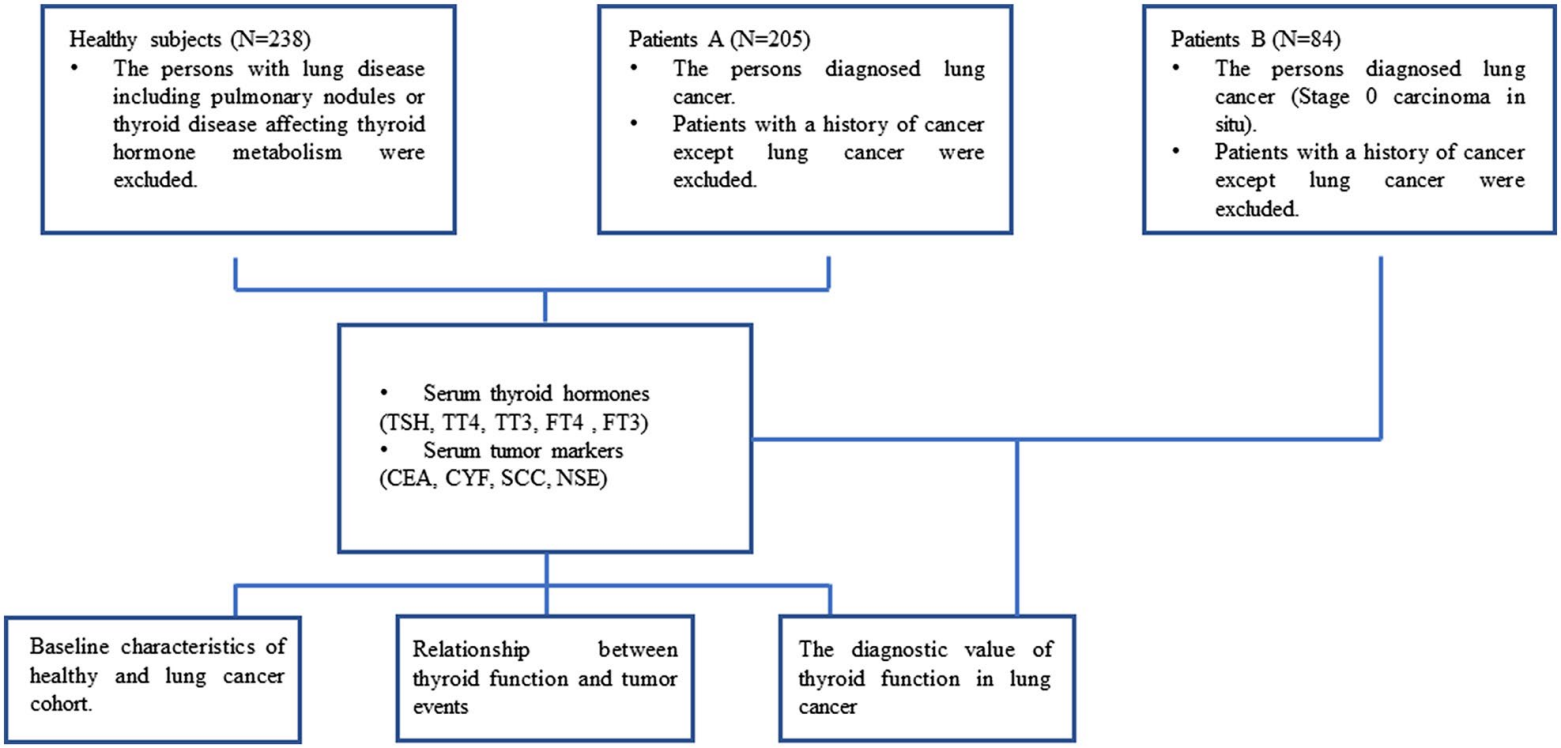
Flow diagram showing the study process.

According to the inclusion and exclusion criteria, a total of 238 healthy subjects and 289 patients were included in our study (Figure 1). In lung cancer patients A group, the mean age of the patients was 66.819 ± 0.655 years (matched to the control group). According to pathological examinations, 154 and 51 patients were diagnosed with non-small cell lung cancer and small cell lung cancer, respectively. Additionally, 179 patients were positive for lymph node metastasis, and 81 patients were classified into stages I + II + III based on the TNM staging system. In lung cancer patients B group, the mean age of the patients was 65.77 ± 0.181 years (matched to the control group). All patients were classified into stages 0 carcinoma *in situ*. Of the 238 healthy subjects, 55 were female and 183 were male, with a mean age of 65.09 ± 0.700 years. The clinical characteristics of the patients and healthy individuals are summarized in Table 1.

### Relationship between thyroid function and tumor events

**Figure 2.**
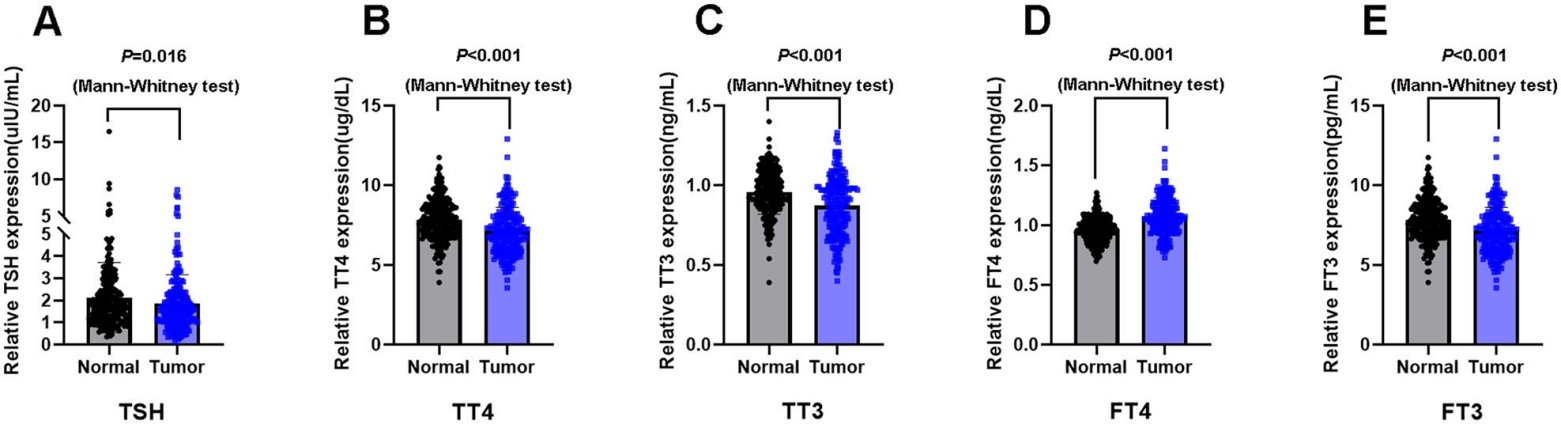
Serum thyroid hormones concentrations in lung cancer patients and their clinical significance. (A) Serum concentration of thyroid stimulating hormone (TSH) in lung cancer patients (*n* = 205) and healthy subjects (*n* = 238). (B) Serum concentration of total thyroxine (TT4) in lung cancer patients (*n* = 205) and healthy subjects (*n* = 238). (C) Serum concentration of total triiodothyronine (TT3) in lung cancer patients (*n* = 205) and healthy subjects (*n* = 238). (D) Serum concentration of free thyroxine (FT4) in lung cancer patients (*n* = 205) and healthy subjects (*n* = 238). (E) Serum concentration of free triiodothyronine (FT3) in lung cancer patients (*n* = 205) and healthy subjects (*n* = 238). Data are shown as the mean ± SD.

Thyroid function markers including TSH, TT4, TT3, FT4, and FT3 were detected. As shown in Figure 2, the concentrations of TSH (*p* = 0.016), TT3 (*p* < 0.001), TT4 (*p* < 0.001), and FT3 (*p* < 0.001) were significantly lower in the lung cancer A group than in the healthy physical examination group. Nevertheless, FT4 levels were increased in patients with lung cancer (*p* < 0.001). To determine the correlation of thyroid function markers with clinical characteristics, lung cancer patients were further subdivided into low-and high-concentration groups according to their median value of thyroid function markers. As indicated in Table 2, low TT3 concentrations were positively associated with age (*p* = 0.010), sex (*p* = 0.010), tumor size (*p* = 0.036), and lymph node metastasis (*p* = 0.022). Moreover, the concentration of FT3 was dependent on age (*p* = 0.002) and tumor size (*p* = 0.003). Serum FT4 concentration was clearly higher in stage IV patients than in stage I–III patients (*p* = 0.001).

**Table 2.** Association of serum thyroid hormones levels with clinicopathological parameters of patients A group with lung cancer.

		Serum TSH level		
		Low	High	
Variables	No of cases	*n* = 129	*n* = 76	*p*
Age (year, mean = 66.8)				0.15
<67	94	64	30	
≥67	111	65	46	
Sex				0.17
Men	159	104	55	
Women	46	25	21	
Tumor type				0.3
SCLC	51	29	22	
NSCLC	154	100	54	
Tumor size (maximum diametercm)				0.82
≤4 cm	93	59	36	
>4 cm	112	70	40	
Lymph node metastasis				0.48
Positive	179	113	69	
Negative	26	16	7	
TMN stage				1.0
I/II/III	81	51	30	
IV	124	78	46	
		Serum TT4 level		
		Low	High	
Variables	No of cases	*n* = 106	*n* = 99	*p*
Age (year, mean = 66.8)				
<67	94	46	48	0.465
≥67	111	60	51	
Sex				
Men	159	85	74	0.350
Women	46	21	25	
Tumor type				
SCLC	51	24	27	0.443
NSCLC	154	82	72	
Tumor size (maximum diametercm)				0.087
≤4 cm	93	42	51	
>4 cm	112	64	48	
Lymph node metastasis				
Positive	179	96	83	0.148
Negative	26	10	16	
TMN stage				0.26
I/II/III	81	38	43	
IV	124	68	56	
		Serum TT3 level		
		Low	High	
Variables	No of cases	*n* = 98	*n* = 107	*p*
Age (year, mean = 66.8)				
<67	94	31	63	0.01
≥67	111	67	44	
Sex				
Men	159	76	83	0.01
Women	46	53	21	
Tumor type				
SCLC	51	21	30	0.274
NSCLC	154	77	77	
Tumor size (maximum diametercm)				0.036
≤4 cm	93	37	56	
>4 cm	112	61	51	
Lymph node metastasis				
Positive	179	91	88	0.022
Negative	26	7	19	
TMN stage				0.836
I/II/III	81	38	43	
IV	124	60	64	
		Serum FT4 level		
		Low	High	
Variables	No of cases	*n* = 107	*n* = 98	*p*
Age (year, mean = 66.8)				
<67	94	54	40	0.166
≥67	111	53	58	
Sex				
Men	159	84	75	0.735
Women	46	23	23	
Tumor type				
SCLC	51	24	27	0.397
NSCLC	154	83	71	
Tumor size (maximum diametercm)				0.665
≤4 cm	93	47	46	
>4 cm	112	60	52	
Lymph node metastasis				
Positive	179	94	85	0.810
Negative	26	13	13	
TMN stage				0.001
I/II/III	81	54	27	
IV	124	53	71	
		Serum FT3 level		
		Low	High	
Variables	No of cases	*n* = 98	*n* = 107	*p*
Age (year, mean = 66.8)				
<67	94	34	60	0.002
≥67	111	64	47	
Sex				
Men	159	76	83	1.0
Women	46	22	24	
Tumor type				
SCLC	51	19	32	0.08
NSCLC	154	79	75	
Tumor size (maximum diametercm)				0.003
≤4 cm	93	34	59	
>4 cm	112	64	48	
Lymph node metastasis				
Positive	179	86	93	0.855
Negative	26	12	14	
TMN stage				0.436
I/II/III	81	36	45	
IV	124	62	62	

### The diagnostic value of thyroid function in lung cancer

**Figure 3.**
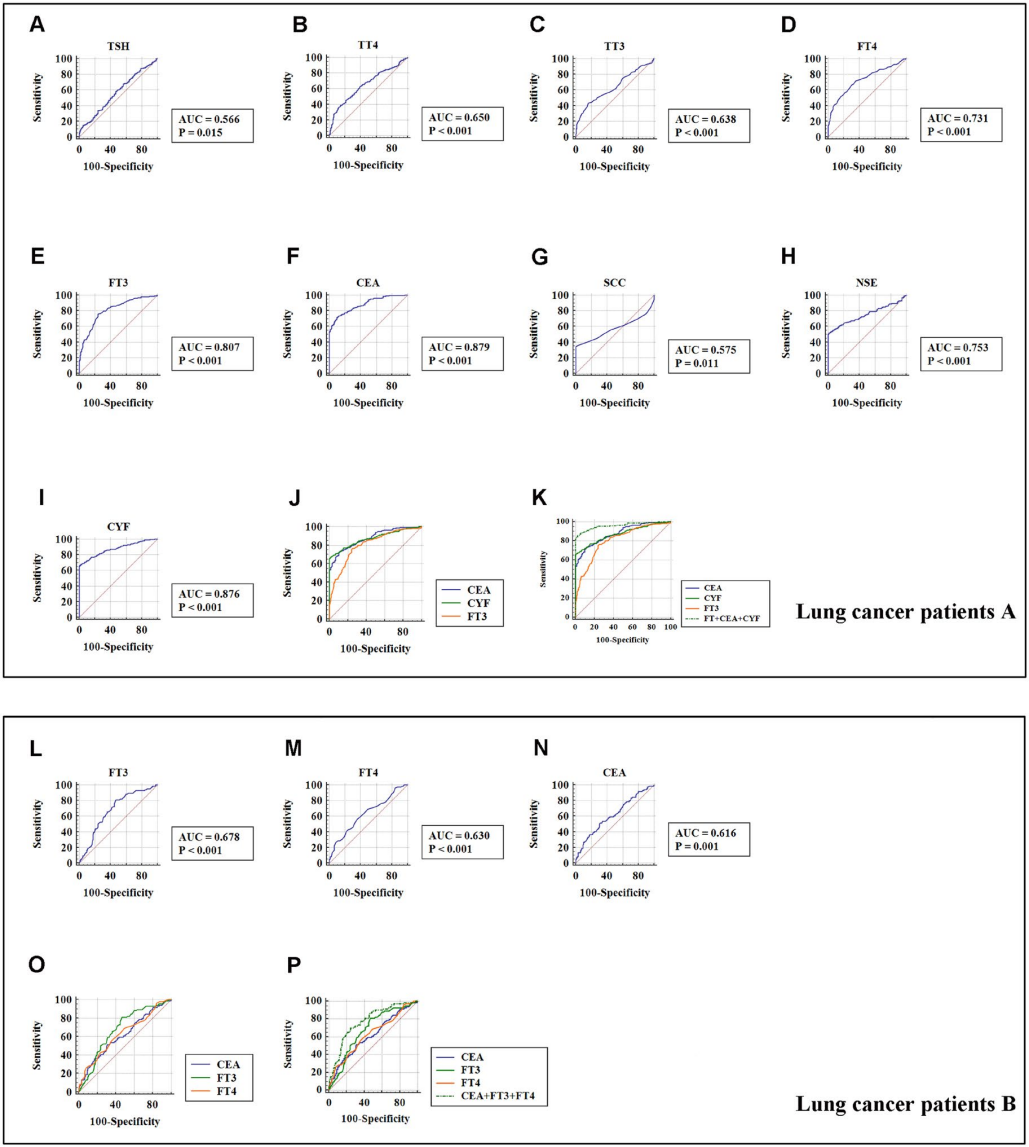
The receiver operating curve (ROC) for the prediction of stage 0 and I–IV lung cancer based on the serum concentration of thyroid hormones and traditional lung cancer markers, using paired healthy subjects as control. (A) ROC curve showing the diagnostic significance of serum thyroid stimulating hormone (TSH) concentration in the lung cancer patients A group. (B) ROC curve showing the diagnostic significance of serum total thyroxine (TT4) concentration in the lung cancer patients A group. (C) ROC curve showing the diagnostic significance of serum total triiodothyronine (TT3) concentration in the lung cancer patients A group. (D) ROC curve showing the diagnostic significance of serum free thyroxine (FT4) level in the lung cancer patients A group. (E) ROC curve showing the diagnostic significance of serum free triiodothyronine (FT3) level in the lung cancer patients A group. (F) ROC curve showed the diagnostic significance of serum carcinoma embryonic antigen (CEA) concentration in the lung cancer patients A group. (G) ROC curve showing the diagnostic significance of serum squamous cell carcinoma (SCC) concentration in the lung cancer patients A group. (H) ROC curve showing the diagnostic significance of serum neuron specific enolase (NSE) concentration in the lung cancer patients A group. (I) ROC curve showing the diagnostic significance of serum CYFRA21-1 (CYF) concentration in the lung cancer patients A group. (J) Comparison of ROC curve showing the diagnostic significance of serum CEA, CYFRA21-1 (CYF) and FT3 concentration in the lung cancer patients A group. (K) Comparison of ROC curve showing the diagnostic significance of serum CEA, CYFRA21-1 (CYF), FT3 and FT3 + CEA + CYF concentration in the lung cancer patients A group. (L) ROC curve showing the diagnostic significance of serum free triiodothyronine (FT3) level in the lung cancer patients B group. (M) ROC curve showing the diagnostic significance of serum free thyroxine (FT4) level in the lung cancer patients B group. (N) ROC curve showed the diagnostic significance of serum carcinoma embryonic antigen (CEA) concentration in the lung cancer patients B group. (O) Comparison of ROC curve showing the diagnostic significance of serum CEA, FT3 and FT4 concentration in the lung cancer patients B group. (P) Comparison of ROC curve showing the diagnostic significance of serum CEA, FT3, FT4 and FT3 + FT4 + CEA concentration in the lung cancer patients B group.

To validate the potential diagnostic utility of thyroid function markers, we firstly performed receiver operating curve (ROC) analysis in stage I–IV lung cancer patients (Figure 3). The area under the curve values (AUCs) of the five thyroid function markers were as follows: 0.566 for TSH (≤1.76 µIU/mL); 0.650 for TT4 (≤6.65 µg/dL); 0.638 for TT3 (≤0.83 ng/mL); 0.731 for FT4 (>1.01 ng/dL); and 0.807 for FT3 (≤2.77 pg/mL). In addition, the AUCs of the four traditional lung cancer markers were 0.879 for CEA (>3.08 ng/mL), 0.575 for SCC (>1.4 ng/mL), 0.753 for NSE (>15.05 ng/mL), and 0.876 for CYF (>3.16 ng/mL). When we included FT3, CEA, and CYF in the diagnosis, the AUC was 0.962 (Figure 3). Particularly, in the lung cancer A group with “normal” CEA levels (0–5.00 ng/mL), 76% (73/96) had low FT3 level (≤2.77 pg/mL). While, in the lung cancer A group with “negative” CEA levels (≤3.08 ng/mL), 76% (41/54) had low FT3 level (≤2.77 pg/mL). Hence, FT3 level could partially improve the detection rate of lung cancer in the patients with “normal” CEA levels or “negative” CEA levels.

**Figure 4.**
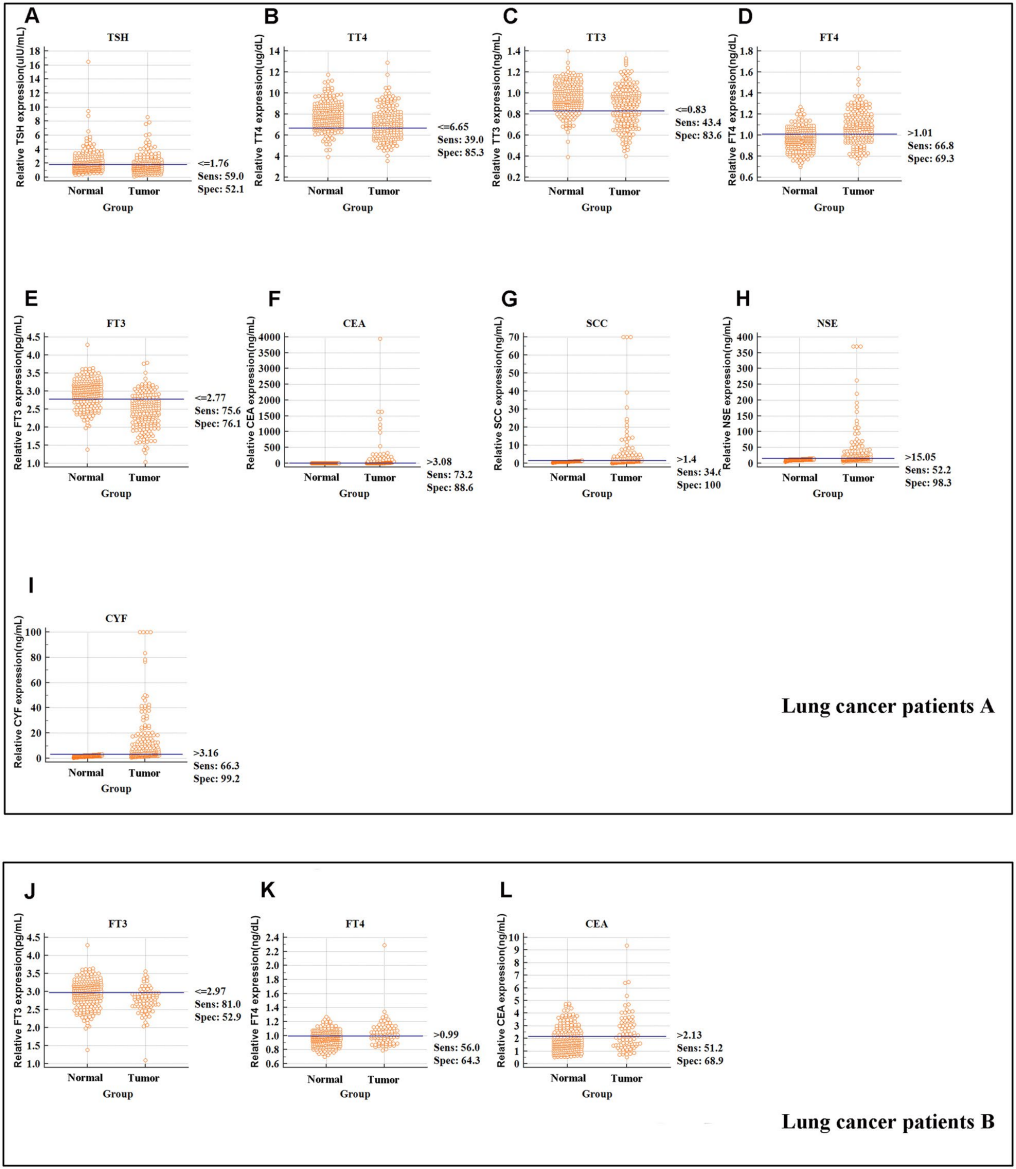
The sensitivity and specificity of markers to differentiate lung cancer patients with different TNM stage from healthy subjects. (A) The sensitivity, specificity and cut-off value of TSH concentration in the lung cancer patients A group. (B) The sensitivity, specificity and cut-off value of TT4 concentration in the lung cancer patients A group. (C) The sensitivity, specificity and cut-off value of TT3 concentration in the lung cancer patients A group. (D) The sensitivity, specificity and cut-off value of FT4 concentration in the lung cancer patients A group. (E) The sensitivity, specificity and cut-off value of FT3 concentration in the lung cancer patients A group. (F) The sensitivity, specificity and cut-off value of CEA concentration in the lung cancer patients A group. (G) The sensitivity, specificity and cut-off value of SCC concentration in the lung cancer patients A group. (H) The sensitivity, specificity and cut-off value of NSE concentration in the lung cancer patients A group. (I) The sensitivity, specificity and cut-off value of CYF concentration in the lung cancer patients A group. (J) The sensitivity, specificity and cut-off value of FT3 concentration in the lung cancer patients B group. (K) The sensitivity, specificity and cut-off value of FT4 concentration in the lung cancer patients B group. (L) The sensitivity, specificity and cut-off value of CEA concentration in the lung cancer patients B group.

Moreover, the sensitivity and specificity of the thyroid function markers were also estimated when they were applied separately or synergistically in the diagnosis of lung cancer. As displayed in Table 3 and Figure 4, the sensitivities of five thyroid function markers (TSH, TT3, TT4, FT3, and FT4) were 59%, 43.41%, 39%, 75.6%, and 66.83%, respectively, and the sensitivities of the four traditional lung cancer markers (CEA, NSE, SCC, and CYF) were 73%, 52.2%, 34.6%, and 66.2%, respectively. Furthermore, the specificities of the five thyroid function markers (TSH, TT3, TT4, FT3, and FT4) were 52.1%, 83.61%, 85.3%, 76.1%, and 69.33%, respectively, and the specificities of the four traditional lung cancer markers (CEA, NSE, SCC, and CYF) were 88.6%, 98.3%, 100%, and 99.2%, respectively. When FT3 were used in combination with CEA and CYF, the sensitivity and specificity of screening for lung cancer were markedly increased to 85.9% and 97.5%, respectively. These results showed that FT3 might distinguish stage I–IV lung cancer cases from healthy controls, especially in patients with “normal” CEA levels.

**Table 3.** The assessment of a biomarker role of the thyroid hormones and traditional lung cancer markers in lung cancer.

Markers	Sensitivity (%)	Specificity (%)	AUC	Stage
TSH	59.00	52.10	0.566	I–IV
TT3	43.41	83.61	0.638	I–IV
TT4	39	85.30	0.650	I–IV
FT3	75.60	76.10	0.807	I–IV
FT4	66.83	69.33	0.731	I–IV
CEA	73	88.60	0.879	I–IV
NSE	52.20	98.30	0.753	I–IV
SCC	34.60	100	0.575	I–IV
CYF	66.20	99.20	0.876	I–IV
CEA + CYF + FT3	85.90	97.50	0.962	I–IV
TSH	65.48	42.44	0.500	0
TT3	84.52	21.43	0.518	0
TT4	64.30	60.90	0.518	0
FT3	81.00	52.90	0.678	0
FT4	56.00	64.30	0.630	0
CEA	51.20	68.90	0.616	0
NSE	73.80	35.70	0.531	0
SCC	26.20	89.90	0.591	0
CYF	40.50	82.40	0.623	0
CEA + FT4 + FT3	70.20	75.20	0.774	0

Furthermore, additional analysis evaluated the diagnostic utility of thyroid function markers in stage 0 lung cancer patients B group. As presented in Table 3 and Figure 4, ROC curve showed that FT3 (≤2.97 pg/mL) and FT4 (> 0.99 ng/dL) level might distinguish stage 0 lung cancer patients from healthy controls with a sensitivity and specificity of 81%, 52.9% and 56%, 64.30%, respectively. When we included FT3, FT4, and CEA in the diagnosis, the AUC was 0.774. The sensitivity and specificity of screening for stage 0 lung cancer were increased to 70.2% and 75.2%, respectively.

## Discussion

The effects of thyroid hormones on human solid tumors are complex, especially for TSH. Several prospective studies have evaluated the association between thyroid hormones and cancer risk. Alf Inge Hellevik et al. found that low thyrotropin concentrations (<0.50 mU/L) were associated with a 1.34-fold increased risk of cancer [15]. However, Samer R. Khan et al. demonstrated that there was a negative correlation between cancer incidence and TSH, but the correlation was not significant [16]. A more recent study conducted by Mariana F. Gayyed showed that a high thyroid hormone receptor α1 expression level was associated with shorter OS of lung squamous cell carcinoma [17]. In this retrospective study, we assessed the relationship between thyroid function and lung cancer. Our results showed that the concentration of FT4 in lung cancer was higher than that in healthy controls, while there was a negative association between other thyroid hormone (including TSH, TT3, TT4, and FT3) concentrations and lung cancer.

In addition, the role of thyroid hormones in lung cancer development has not yet been clearly elucidated. *In vitro*, T4 at physiologic free hormone concentrations and T3 at supraphysiologic concentrations stimulated cell proliferation and increased PCNA abundance in lung cancer cells [18]. This phenomenon can be responsible for an integrin αvβ3-dependent mechanism and be abrogated by the αvβ3 inhibitor Tetrac. Further mechanistic research showed that T4 and T3 bind αvβ3, leading to the activation of the MAPK/ERK1/2 or PI3K-AKT pathway, followed by the upregulation of p21, HIF-1a, Ndrg2, and so on [19]. Recent *in vivo* research has confirmed that treatment with only T4, but not T3, promoted lung cancer growth in an orthotopic mouse model [20]. We extended the existing evidence regarding the important role of thyroid hormones in lung cancer by analyzing the association between thyroid hormones and clinicopathologic characteristics of lung cancer. It was shown that the enhancement of FT4 tended to be greater in the more advanced cancer stage, while low T4 concentration might occur in elderly and more cancer mass patients. Although we enrolled two groups that were matched by age and sex, the limitations of our study are its retrospective nature. Further prospective studies are needed to confirm the association between thyroid hormone levels and the clinicopathological features of lung cancer patients.

Early screening is still an effective approach for decreasing the mortality of lung cancer. To date, the search for a reliable and non-invasive biomarker of lung cancer has been unsuccessful. In the present study, we evaluated the diagnostic value of thyroid hormone levels in lung cancer. Remarkably, in stage I–IV lung cancer patients, the ROC analysis indicated that FT3 had potential diagnostic efficiency for lung cancer with a high AUC of 0.807, respectively. Furthermore, the combination of thyroid function markers (FT3) and traditional lung cancer markers (CEA and CYF) might be useful for the efficient diagnosis of lung cancer, with an AUC of 0.962. While, in stage 0 lung cancer patients, the combination of these markers (FT3, FT4 and CEA) showed a higher detection capacity, suggesting that their application as a panel might allow for the early detection of lung cancer.

In conclusion, compared with healthy people, the concentrations of TSH, TT3, TT4, FT3, and FT4 were significantly abnormal in patients with lung cancer. The concentrations of TT3 and FT3 were associated with age and tumor size. Meanwhile, serum FT4 concentration is related to the TNM stage. More importantly, FT3 might act as biomarkers for the diagnosis of lung cancer. Nonetheless, there are some limitations that should be acknowledged. First, this study was retrospective and had a limited sample size. Second, the underlying molecular mechanisms of THs and TRs in lung cancer remain uncertain, and future *in vitro* studies must be done.

## Data Availability

Original data will be provided upon request after removal of personal identifiers.
